# Nordic Society for Radiation Protection—an important forum for radiological protection knowledge

**DOI:** 10.1093/rpd/ncaf076

**Published:** 2025-08-28

**Authors:** Jack Valentin, Sören Mattsson

**Affiliations:** KcRN (Swedish Radiation Emergency Medicine Centre), Cancercentrum Karolinska, R8:03, Visionsgatan 56, SE-171 64 Solna, Sweden; Jack Valentin Radiological Protection, Öregrundsgatan 15, SE-115 59 Stockholm, Sweden; Medical Radiation Physics Malmö, Department of Translational Medicine, Lund University, Skåne University Hospital, SUS Malmö, SE-205 02 Malmö, Sweden

## Abstract

The Nordic Society for Radiation Protection (NSFS) was founded in 1964 at the initiative of Rolf Sievert. Its task is to activate the exchange of knowledge and experience in the Nordic countries regarding protection against ionizing and non-ionizing radiation, for all kinds of occupational, medical, or public exposures. NSFS has always included members from all five Nordic countries and was a founding member of IRPA, the International Radiation Protection Association. Since 1966, NSFS has had regular meetings at 3- or 4-year intervals, in turn in each of the Nordic countries. In addition, NSFS has arranged various themed meetings. The meetings of the Society have been informal and collaborative and important for transfer of skills between generations. The activities have stimulated Nordic co-operation regarding nuclear safety research, nuclear waste, radioecology, medical radiology, and clinical physics, as well as Nordic postgraduate courses. NSFS lives up to the IRPA motto of being the international voice of the RP profession.

## Introduction

The Nordic Society for Radiation Protection (NSFS) was founded on 10 June 1964, at the initiative of Rolf Sievert, who was then the head of SSI, the Swedish Radiation Protection Authority [1]. It has the task of activating the exchange of knowledge and experience in the Nordic countries regarding protection against ionizing and non-ionizing radiation, both in terms of occupational exposures, exposures of patients for diagnostic or therapeutic purposes, and exposures of members of the public (see the Society’s statutes [2]. This article will highlight some instances when NSFS contributed to important developments in radiological protection.

## History of NSFS

### The organizational context around 1965

The early 1960s were a period of amazing developments in radiological protection. Since World War II and its conclusion with the use of ‘atomic’ bombs, the use (and misuse) of radioactive materials had increased enormously [3]. The peaceful use of nuclear power had only just started, with the first commercial nuclear power plant having been connected to the grid in August 1956 (Calder Hall in the UK, although USA and Russian sources also claim to have had the first commercial plants in their countries). The first nuclear accidents with off-site consequences occurred in 1957 (Windscale in the UK, and Kyshtym in the then Soviet Union, which was however publicly revealed and described only many years later).

Given this indiscriminate growth of the use of ionizing radiation and concomitant accidents and ‘over-exposures’, it is not surprising that the number of international organizations with an interest in this topic grew rapidly [3]. ICRP, the International Commission on Radiological Protection, and ICRU, the International Commission on Radiation Units and Measurements, both of which were first conceived at the 1st International Congress of Radiology in London 1925, had both been dormant during the world war, but were revived in 1950 at the 6th Congress, again in London [4].

UNSCEAR, the United Nations Scientific Committee on the Effects of Atomic Radiation, was created in 1955 through a resolution of the UN General Assembly to assess levels and effects of exposure to ionizing radiation. In 1953, US President Dwight Eisenhower addressed the UN General Assembly with his famous ‘Atoms for Peace’ call for furthering peaceful uses of nuclear energy and ensuring that such energy should not serve any military purpose. That speech led to the establishment, in 1957, of IAEA, the International Atomic Energy Agency.

In addition to such truly international organizations, several regional organizations were also formed, including, e.g. EURATOM, the European Atomic Energy Community (1958), and NEA, the Nuclear Energy Agency of the OECD (1958). These were followed in the late 1960s by the Foro Iberoamericano (Spain, Portugal, and Latin American countries) and CRCPD, the (US) Conference of Radiation Control Program Directors.

Thus, there were lots of organizations that issued technical advice of varying degrees of legal validity and regulatory force. However, particularly at that time, none of the organizations mentioned above was ‘open’ to individual membership or participation in their work, nor even just passive presence as an audience at meetings. Therefore, there was a need for a way for radiation users and specialists in different countries to join forces and act in a professional context.

### The health physics society and the birth of IRPA

This need for an international professional society was clearly perceived by Karl Ziegler Morgan, the forceful chairman of HPS, the Health Physics Society in the US, which was the very first organization for radiological protection professionals. That society had its origins in the Manhattan Project, which involved work with quantities and types of radiation and radioactive materials at a scale that had never before been imagined [5]. Consequently, a number of participants in the Project were assigned full-time to what was termed ‘health physics’, thus becoming the very first representatives worldwide of our profession. The first formal training programme was initiated in 1944 at what is now ORNL, the Oak Ridge National Laboratory. Elda Emma Anderson, a former Manhattan Project participant, took over the ORNL training programme in 1949 and was an ardent proponent of the idea that ‘health physics’, i.e. radiological protection, was a true profession that would justify the formation of a professional society. Elda Anderson organized the first international course in health physics in Stockholm in 1955 and later on similar courses in Belgium in 1957 and Mumbai, India, in 1958 [6].

Largely at Elda Anderson’s initiative, HPS was formally constituted in 1955 and Morgan was elected its first president. From 1957, Morgan noted that an increasing number of people from outside the USA applied for membership. In 1963, with more than 100 overseas members from 17 different countries, HPS decided to start in earnest a process to initiate an international association, and Morgan was given the task to carry out these ideas. Early in 1964, the enthusiastic Morgan wrote a thousand letters to experts in different countries, including Rolf Sievert in Sweden. Morgan’s proposals were favourably received in many quarters, but Sievert (who had at one time imagined that ICRP might develop into such an organization) was initially apprehensive [1].

Morgan’s efforts generated an *ad hoc* Committee meeting in London in February 1964, which advocated an international society with national or regional societies as members. A provisional society with a *pro tempore* Executive Council was formed in 1964, and the organization, IRPA (the International Radiation Protection Association) was formally established in Rome, at its first Congress, in 1966.

The only problem with that idea was that there were very few such societies that could aspire to become members of the new international society (one exception being the Society for Radiological Protection in the UK, which had been formed in 1963). However, the appropriate wheels to organize such national societies were set in motion in several European countries, and Sievert, who was capable when in the right mood of an enthusiasm matching Morgan’s, managed to get 45 names, including his own, onto a circular proposing the formation of a Nordic Society for Radiation Protection, Nordiska Sällskapet för Strålskydd (NSFS), and organized a first meeting in the spring of 1964.

### The early years of NSFS

At the inaugural meeting in Stockholm, there were 8 Danes (including E. Juel Henningsen), 5 Finns (including J. K. Miettinen), 1 Icelander (G. F. Petersen), 5 Norwegians (including K. Koren), and 34 Swedes (including Sievert). Right from the start, the society thus included members from all five Nordic countries [7].

It is also noteworthy that the heads of the regulatory authorities in the Nordic countries chose not just to support their staff as members and participants, but also to participate personally. This remarkable confidence in the importance of the Society and its meetings has been a hallmark of NSFS and has certainly contributed to its scientific and organizational success. It also reflects the fact that the heads of the authorities were experts in their area, selected by their governments for their radiological protection competence, not just for being ‘good managers’.

NSFS participated in the 1964 *pro tempore* meeting of IRPA and became a formal member of IRPA at the first possible occasion, in 1965, even before IRPA had been formally constituted. The Society shares this honour with the corresponding societies in the USA, France, Luxembourg, Belgium, Japan, UK, Israel, Italy, and the Netherlands.

### Some highlights from earlier NSFS meetings

Before 2023, NSFS had 18 regular full meetings [8] (usually under a specific scientific theme, such as radioactive waste or natural background radiation). The Society has also had a number of themed other meetings, e.g. on radon (Geilo, Norway, 1980), on radiological protection in nuclear installations (Öland, Sweden, 1985), and on quality in radiological protection work (Malmö, Sweden, 2001 and 2004).

The Society has always benefited from the strong commitment of members who valued the Society and worked hard to achieve a good collaboration climate and overcome any obstacles due to language barriers or political differences between the member countries. As one of many good examples, members often refer to the late Tua Rahola from the Finnish regulator, STUK.

The second full meeting of NSFS took place in 1968 in Oslo, Norway, at Voksenåsen under the chairmanship of Kristian Koren. The ‘Farmer F-N risk curve’ [9] was discussed at the meeting, but the place is perhaps most famous in radiological protection circles since during an ICRP meeting there a few years later, John Dunster saved Henri Jammet from drowning in the hotel pool.

In 1971, NSFS met in Copenhagen. True to Nordic traditions of non-adversarial interactions and giving everyone a chance to be heard, the programme committee had invited the very controversial Dr Ernest Sternglass as a guest speaker. His presentation on infant mortality, supposedly related to releases of radionuclides from nuclear installations, attracted a great deal of interest but in the discussion, he was accused of the heinous crime of cherry-picking results that seemed to support his hypothesis and discarding any data that seemed to contradict it. The Nordic participants at the meeting presented an impressive range of talks on all aspects of radiological protection.

The 1977 meeting of NSFS took place in Visby on the Swedish island of Gotland. Natural background radiation and radon problems were discussed and the meeting and that theme received good press coverage.

An important theme meeting of NSFS, at Hanstholm in Denmark in 1983, was dominated by a presentation by Bo Lindell on the philosophy of optimisation of protection using cost–benefit analysis. Eric E. ‘Bill’ Pochin from ICRP was also present, which may have spurred Lindell to stress that he did not support the UK NRPB idea [10] of adding a ß term to the optimisation equation (representing the number of people and the highest doses, thus reflecting subjective risk perceptions).

The next general meeting of NSFS, in Copenhagen, Denmark, 1984, had Robin Mole from the UK as a guest speaker, talking about injuries caused if foetuses were irradiated.

NSFS also began to collaborate with its sister society for German-speaking countries, the Fachverband für Strahlenschutz, which had built up a tradition of ‘Inseltagungen’ (meetings on islands). A first such joint meeting took place in Mariehamn on Åland (Finland) in 1987. A following joint meeting, in 1989, again took place in Visby, with Lindell as Chair and Jack Valentin as joint ‘Tagungssekretär’ and primary liaison with the Fachverband. The Congress Dinner was much appreciated by most participants but perhaps not so much by the well-known German anti-nuclear activist, Dr Inge Schmitz-Feuerhake, who was sitting at the same table as Dr Jaak Sinnaeve, who was in charge of the vast research funds that the European Commission could grant for radiation and nuclear issues. Sinnaeve had a great interest in radiological protection and was an ardent supporter of ICRP, but during the dinner, he expressed some politically very conservative views on Europe and its future interaction with developing countries.

In 1990, NSFS met at Ronneby in Sweden. Abel González of IAEA and Burton Bennett of UNSCEAR were invited guests. Although both of them made interesting scientific presentations, the participants’ lasting memory of the meeting must be Bennett’s long and humorous dinner speech, made to everybody’s astonishment in broken but grammatically flawless Swedish! An important theme during the discussions was risk and the question of whether it would be feasible to limit maximum consequences, regardless of their low probability (which would probably exclude any use of nuclear power).

### Important NSFS contributions to Nordic policy

It is self-evident that a forum where practitioners from different areas and regulators can meet and freely exchange views and ideas will lead to knowledge transfer and improved practices and methods. But some specific observations may also be worth mentioning.

When NSFS was created, it quickly became a framework for formal co-operation between the Nordic radiological protection authorities. At that time, no similar framework existed for the emerging reactor safety regulators [11]. Efforts to create such a framework led to what is now, after various permutations, the very successful NKS (Nordic nuclear safety research).

At a joint meeting in 1977, the directors of the radiological protection and the nuclear safety authorities established a permanent Nordic forum, which came to be named ‘the Chiefs’ group’. A tradition was soon established that this group would always meet in connection with the regular NSFS meetings. Furthermore, the Chiefs’ group was also engaged in a series of Nordic-British meetings (which the Brits slightly quaintly called the Anglo-Nordic meetings). These would involve contacts not with the UK regulators but primarily with NRPB (i.e. in current terminology, the support organization, corresponding in the Nordic countries usually to the research departments of the regulatory authorities, while separate support organizations were not the norm in the Nordic countries).

NSFS certainly influenced the scope of regulatory activities in the Nordic countries. For instance, non-ionizing radiation became an integral part of the meetings from the early 1970s and was soon integrated into the pertinent legislation. The approach to natural background radiation, first from radon but then more generally, was also influenced by NSFS, and regulatory attitudes shifted towards exploring possible ways of formal dose limitation in such contexts.

Policies and ideas regarding radioactive waste often emerged at first from NSFS activities and meetings. NSFS members and the ideas presented at meetings also tended to find their way into the series of ‘Flag books’, joint guidelines and recommendations issued by the Nordic regulatory authorities on topics such as the application and interpretation of general or topical ICRP recommendations.

NSFS also prompted the regulators to facilitate the organization of Nordic seminars (notably on radioecology) and PhD study courses.

## Summary and highlights from the 2023 Malmö meeting

The meeting had attracted 113 participants from 11 countries. It started with the ‘Bo Lindell lecture’ with the title ‘Trends in collective effective doses from radiological procedures’ given by Ritva Bly from Finland. There were 58 oral presentations and 26 scientific posters. There were also approximately 10 presentations by sponsors for the meeting and 6 posters from related societies. Invited presentations were given by the IRPA President Bernard Le Guen and the ICRP Chair Werner Rühm, who talked about the forthcoming set of ICRP Recommendations. In that context, we mentioned that NSFS needs to form a view on the handling of exaggerated low-dose risks—either the ‘European’ approach to advocate more common sense in clearance criteria [12], or the ‘American’ approach to postulate thresholds below which no adverse effects of radiation are expected [13].

Vadim Chumak described the R/N situation in Ukraine and possible consequences, the meeting discussed AI in radiological protection, future competence supplies of radiation professionals, and a special session was organized by and for young radiation professionals.

**Figure 1:**
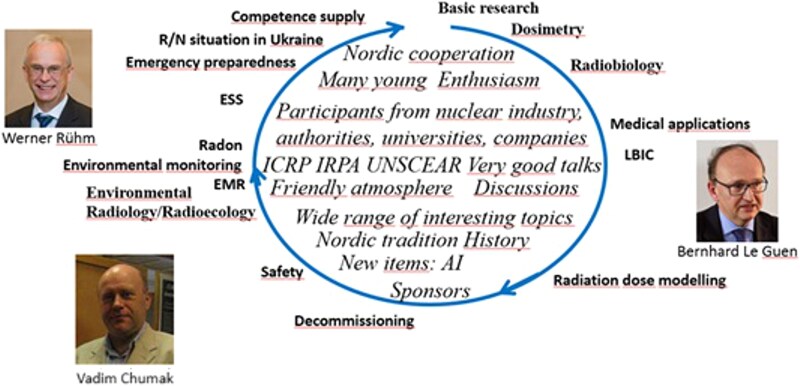
The authors’ impression of the meeting and the topics discussed. ESS = European Spallation Source; EMR = Electronic Medical Record; LBIC = Lund Bioimaging Centre.

Figure 1 shows some of the topics that were discussed at the conference while also trying to characterize it.

IRPA’s Task Group on mentorship was described and discussed. All experienced professionals have to face the issue of how to best convey the knowledge we have gained to recently hired radiation protection staff.

At the meeting, information was given on European co-operation in radiation protection research and ongoing Nordic projects on shielding and dose constraints. The co-operation between the Nordic radiation protection authorities was also highlighted. The radiation protection problems related to high radon levels were also brought to the fore with reference to ongoing research projects in Finland, Greenland, and Canada.

In a session on radiological protection and risks around ESS (the European Spallation Source located close to Lund), source term, accident analysis, detection limits for ESS radionuclides in the environment, external and internal doses at accidents and related risks were discussed.

Regarding radiation dosimetry, lectures around dosimetry during radiotherapy, patient vs staff radiation doses, the dose calculation programme IDAC-BioDose and a new app for ICRP’s dose data were given.

In the field of radiation biology, lectures were presented on non-targeted effects, apoptosis, genome sequencing, mixed exposures, and the importance of *in vivo* measurements.

The balance between risk and benefit was discussed in various contexts, including occupational exposures, the use or non-use of lead aprons, and in connection with the release of patients and waste handling.

The protection of life, health, the environment, and other important societal interests after a nuclear detonation, as well as emergency preparedness in general, was highlighted with recommendations for repeated exercises, making full use of existing measuring instruments such as liquid scintillators and gamma cameras as well as decontamination methods. AI for gamma spectrometry was also discussed. Capacity regarding biodosimetry was urgently called for. There may be a need for a Nordic initiative.

In a number of lectures, ongoing research in environmental radiology was highlighted: ^14^C, algae as bioindicators, radionuclides in wood and seafood, effects of climate change, pit lakes, natural radionuclides in the Arctic, release of ^137^Cs and ^210^Po at forest wildfires, contamination in the Gävle area, the SFR waste repository, and modelling of redistribution of fallout.

Within medical applications, contributions about intravascular radiotherapy, calibration for radiotherapy dosimetry, dose to patient vs dose to staff, and NaCl for patient dosimetry were presented. Information around ongoing decommissioning at Risö and about Swedish nuclear waste was also given.

The final discussion was ended by a wish-list for the next NSFS XX conference in Norway:

- Bio-dosimetry
- Risk–benefit in medical imaging
- New nuclear reactors, SMR
- Ageing reactors
- Role of NSFS in radiation protection
